# MYCL1 Amplification and Expression of L-Myc and c-Myc in Surgically Resected Small-Cell Lung Carcinoma

**DOI:** 10.3389/pore.2021.1609775

**Published:** 2021-06-18

**Authors:** Jing Qin, Fajun Xie, Chenghui Li, Na Han, Hongyang Lu

**Affiliations:** ^1^ Department of Thoracic Medical Oncology, Cancer Hospital of the University of Chinese Academy of Sciences (Zhejiang Cancer Hospital), Institute of Basic Medicine and Cancer (IBMC), Chinese Academy of Sciences, Hangzhou, China; ^2^ Zhejiang Key Laboratory of Diagnosis and Treatment Technology on Thoracic Oncology (Lung and Esophagus), Zhejiang Cancer Hospital, Hangzhou, China

**Keywords:** immunohistochemistry, small-cell lung carcinoma, C-MYC, fluorescence *in situ* hybridization, MYCL1 amplification, L-myc

## Abstract

**Purpose:** The Myc family, especially *C-MYC* and *MYCL1*, has been found involved in small-cell lung carcinoma (SCLC). Identification of the frequency of C-MYC and MYCL1 expression among SCLC patients may help to identify potential targets for therapeutic intervention. Our aim was to detect *MYCL1* amplification, L-Myc and c-Myc expression, and investigate clinicopathological characteristics and survival status in patients with surgically resected SCLC.

**Methods:** *MYCL1* amplification was detected using fluorescence *in situ* hybridization (FISH), while L-Myc and c-Myc protein expressions were determined using immunohistochemistry (IHC) in the primary tumors of 46 resected SCLC patients.

**Results:** Among the 46 evaluated specimens, *MYCL1* amplification was identified in 3/46 cases (6.5%). One of the positive cases was *MYCL1* gene amplification combined with fusion. 3/46 (6.5%) was positive for L-myc protein expression, and 4/46 (8.7%) was positive for c-Myc protein expression.

**Conclusion:** Our study firstly multidimensional explored the expression of *MYCL1* amplification, L-Myc and c-Myc protein and investigated clinicopathological characteristics and survival status in patients with surgically resected SCLC, which makes a contribution to subsequent research and therapeutic strategies.

## Introduction

Lung cancer is a leading cause of cancer-related death globally and domestically [1, 2]. Small-cell lung carcinoma (SCLC), accounting for about 15% of all lung cancers, is considered as an aggressive neuroendocrine lung cancer and has a high propensity for early development and extensive metastatic dissemination. SCLC is a lethal disease characterized by rapid recurrence and dismal prognosis, and there is no other effective treatment option except chemotherapy and radiation. The median overall survival (OS) for patients with limited-disease and extensive-disease SCLC is 15–20 months and 8–10 months, respectively [3]. Apart from the recent advances in immunotherapy, the medical management of SCLC has changed little over several decades [4]. Hence, it is crucial to explore novel molecular targets activated by genetic alterations in SCLC.

Recently, the whole-genome profiling has been used to obtain information about the alterations of the activated genes in SCLC. The amplification of Myc family oncogenes *MYCL1* (1p34), *MYCN* (2p24), and *C-MYC* (8q24) was detected frequently for mutually exclusive interactions [5]. Currently, Ireland et al*.* have showed that *C-MYC* drived SCLC subtype evolution *via* reprogramming neuroendocrine peculiarity, which revealed a conserved trajectory from neuroendocrine to non-neuroendocrine subtypes, and a molecular evolution from ASCL1^+^ to NEUROD1^+^ to YAP1^+^ subtypes [6]. Hwang et al*.* have explored *C*-*MYC* amplification by chromogenic *in situ* hybridization (CISH), and it was identified in 9% of SCLCs and was correlated with protein expression. Regrettably, 83% cases were biopsies and only 17% cases were larger specimens [7]. In addition, the role of MYCL1 remains unclear in SCLC. MYCL1 exerts weaker effects than c-Myc in cell growth, apoptosis and transformation [8]. However, it makes the reprogramming of fibroblasts into the induced pluripotent stem cells [9] and L-Myc expression by dendritic cells is required for optimal T-cell priming [10]. *MYCL1* is amplified and overexpressed in some malignancies [11]. L-Myc acts as a transcription factor and is targeted by the transcription factor achaete-scute homolog-1 (ASCL1), which plays crucial roles in promoting the progression of SCLC [12]. Xiong, F et al*.* have found that single-nucleotide polymorphism (SNP) of an intron of *MYCL1* was associated with the susceptibility to SCLC [13]. The amplification and expression of MYCL1 were observed in a majority of human SCLC cell lines [14]. However, correlations of MYCL1 and C-MYC with clinicopathological characteristics and outcomes in SCLC remain largely unknown.

Hence, the goal of this study was to detect *MYCL1* amplification, L-Myc and c-Myc expression, and investigate clinicopathological characteristics and survival status in patients with surgically resected SCLC in China.

## Materials and Methods

### Study Population

We retrospectively collected 59 consecutive patients with resected SCLC at Cancer Hospital of the University of Chinese Academy of Sciences (Zhejiang Cancer Hospital) in China (Hangzhou) between April 2008 and November 2016. The pathological diagnosis of SCLC was based on the standard criteria defined by the World Health Organization (WHO) [15]. The tumor stage was classified according to the tumor-node-metastasis (TNM) classification for lung cancer, eighth edition [16]. Eight patients who experienced neoadjuvant chemotherapy or chemoradiotherapy were excluded from our study due to disturbed effects of treatment-induced DNA damage. Five cases were removed because of insufficient tissue remaining. Therefore, a total of 46 patients with SCLC who underwent surgery were enrolled in the present study. Formalin-fixed paraffin-embedded (FFPE) blocks from 46 SCLC patients were retrospectively collected. Tumor tissues were taken from surgically resected tumors. The clinical characteristics (gender, age, smoking status, tumor stage, lymph node metastasis and brain metastasis) were obtained from the medical records (Table 1). This study was approved by the medical Ethics Committee of Zhejiang Cancer Hospital.

**TABLE 1:** Clinical characteristics of 46 SCLC patients.

Characteristics	Cases (n)
Sex	
Male	36
Female	10
Age	
<60	24
≥60	22
Smoking	
Non-smoker	13
Light smoker (≤10 pack-years)	3
Moderate smoker (10–20 pack-years)	6
Heavy smoker smokers (≥20 pack-years)	24
Stage	
IA	17
IB	5
IIA	0
IIB	9
IIIA	14
IIIB	1
Lymph node metastasis	
N0	22
N+	24
Brain metastasis	
NO	34
YES	12

### Immunohistochemistry

IHC was performed on FFPE tumor tissues using anti-L-Myc antibody (ab28739, Abcam Inc. Cambridge, MA, United States) and anti-c-Myc antibody (ab32072, Abcam) as per the manufacturer’s instructions. The anti-L-Myc antibody (ab28739) consists of rabbit polyclonal to L-Myc. The anti-c-Myc antibody (ab32072) is a rabbit monoclonal antibody and is specific for the endogenous c-Myc.

Tumor specimens were fixed with 10% formalin and embedded in paraffin, followed by deparaffinization, rehydration, endogenous peroxidase blocking, and antigen retrieval. Then the specimens were blocked with 1% bovine serum albumin (BSA) for 10 min at room temperature and incubated with anti-L-Myc antibody (ab28739, 1:200, Abcam) or anti-c-Myc antibody (ab32072, 1:100, Abcam) overnight at 4°C, followed by incubation with iVisionTM Poly-HRP Sheep anti-rabbit secondary antibody (DD13030, Ascend Bio. purchased by Xiamen Talent Biomedical Technology Co., Ltd. People’s Republic of China) for 1 h at 37°C. Next, 3,3′-diaminobenzidine (DAB) kit (KS-003030, Ascend Bio.) was used to visualize the immunoreactivity. PBS was used as negative control instead of primary antibody. Each slide was evaluated independently by two pathologists who were blinded to clinical information. Interpretation of L-Myc expression level: if ≥ 10% cells showing brown color were defined as “positive expression”; No staining or positive staining in less than 10% cells was defined as ‘‘negative expression”. Interpretation of c-Myc expression level: if ≥40% cells showing brown color were defined as “positive expression”; No staining or positive staining in less than 40% cells was defined as “negative expression”. When different interpretations occurred, slides were reviewed until consensus was obtained. Results interpretation referred to the published paper [11,17].

### Fluorescence *in situ* Hybridization

FISH was performed on FFPE tumor tissues using MYCL1 Break Apart Probe (Empire Genomics LLC, purchased by Genediagnostic. Inc. People’s Republic of China) according to the manufacturer’s instructions.

The MYCL1 Break Apart Probe consists of DNA labeled with Spectrum Green and Spectrum Orange. The DNA probe hybridizes to chromosome 1p34.2 in normal metaphase spreads and interphase nuclei.

The FFPE sections (4–5 μm) were deparaffinized, treated with Pretreatment Solution Citric at 98°C, and digested in pepsin solution. The probe (10 μl) was added to each slide. The target DNA and probes were co-denatured at 83°C for 5 min and incubated in a humidified hybridization chamber at 37°C overnight, followed by three post-hybridization washes in 1× wash buffer A at 37°C, 5 min each. Finally, the slides were air-dried and counterstained with 4’,6-diamidino-2-phenylindole (DAPI)/antifade solution. Signals for each locus-specific FISH probe were assessed under an Olympus BX51 microscope (Olympus Corp.). FISH results were evaluated by two pathologists with close correlation regarding the site of interest in FISH analysis with histomorphology in hematoxylin-eosin (HE) stained slides.

The MYCL1 FISH break-apart results were based on at least 100 evaluable tumor cell nuclei. A break-apart/split FISH signal was considered as abnormal in case of “orange and green split-signals” with a clear-cut distance of at least two signal diameters. A cut-off of at least 15% [mean +3 standard deviation (SD)] “break-apart” events was used as the threshold for MYCL1 FISH-abnormal.

The MYCL1 FISH amplification results were based on at least 100 evaluable tumor cell nuclei. The number of orange-green fusion signals in each nucleus was counted and calculated. A cut-off of 2.25 (mean + 3SD) signals on an average was used as the threshold for MYCL1 FISH-abnormal.

### Follow-Up

The follow-up deadline was February 10, 2020. Among all the patients, 21 patients were alive, and 25 patients were dead. The survival time was calculated from the date of pathological diagnosis.

### Statistical Analyses

Statistical analysis was performed using SPSS 15.0 statistical software (SPSS Inc. Chicago, IL, United States). All data were presented as mean ± SD.

## Results

### Prevalence of *MYCL1* amplification and Fusion by FISH

**FIGURE 1:**
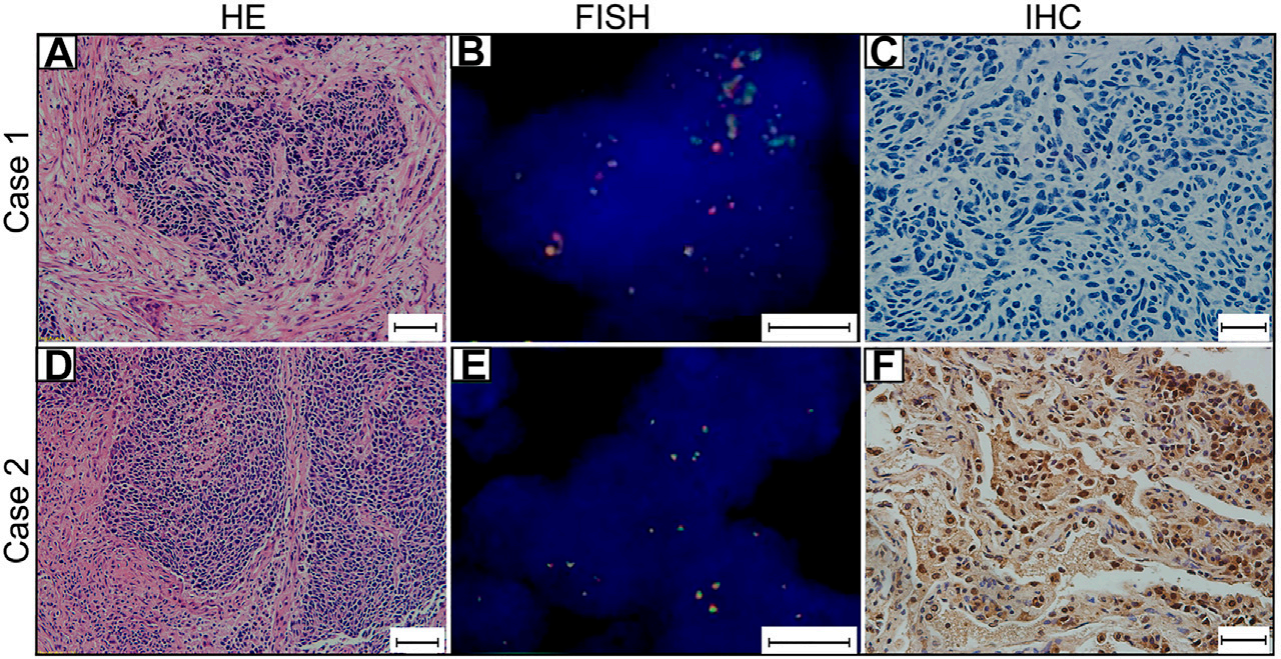
*MYCL1* Fluorescence *in situ* hybridization (FISH) and L-Myc Immunohistochemistry (IHC) in small-cell lung carcinoma (SCLC). Case 1 represents *MYCL1* amplification patient. **(A)** The representative image of hematoxylin and eosin (HE) staining (scale bar 50 μm). **(B)** *MYCL1* amplification by FISH (scale bar 10 μm). Green spectrum denotes DNA 3′-end and Orange spectrum denotes DNA 5′- end. **(C)** L-Myc protein expression negative (scale bar 20 μm). Case 2 represents L-Myc protein expression positive patient. **(D)** The representative image of HE-staining (scale bar 50 μm). **(E)** *MYCL1* non-amplification (scale bar 10 μm). **(F)** L-Myc protein expression positive (scale bar 20 μm). Brown color of the nucleus was defined as positive staining.

**FIGURE 2:**
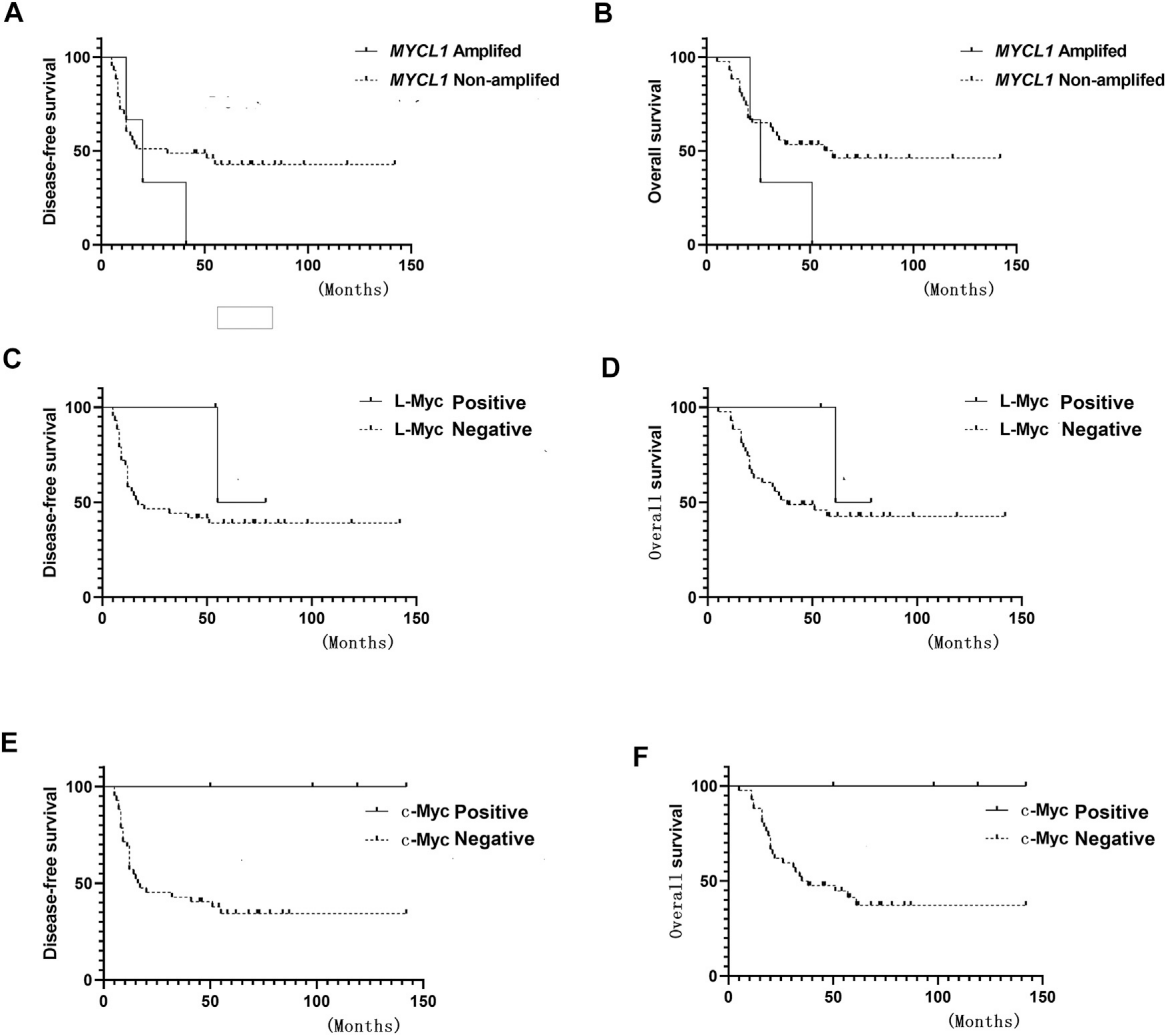
Survival curve **(A)** Disease-free survival (DFS) of *MYCL1* amplification **(B)** Overall survival (OS) of *MYCL1* amplification **(C)** DFS of L-Myc protein expression **(D)** OS of L-Myc protein expression **(E)** DFS of c-Myc protein expression **(F)** OS of c-Myc protein expression.

*MYCL1* amplification and fusion were evaluated by FISH in 46 SCLC specimens. As shown in Figure 1, case1 represented *MYCL1*amplified patient. According to our results, three cases showed *MYCL1* gene alterations, where two were amplification and the other was amplification combined with fusion. *MYCL1* gene alterations were identified in 3 of 46 cases (6.5%). Three amplified cases were positive for high *MYCL1* gene copy number, 17, 5.45, and 5.7 per nucleus, respectively. It was demonstrated that these *MYCL1* gene alterations were caused by the increased gene copy number gain on the chromosome. In addition, the ratio of isolated signal cells was 53.8% in the case with *MYCL1* fusion. Of the three positive patients, their ages were more than 60 years old. Two male patients had moderate and heavy smoking history staged with IA and IIB respectively, and one female patient without smoking exposure staged with IIB. No brain metastasis was observed in the above three positive cases during the follow-up (Table 2). All three positive patients had died. The DFS time of these three cases was 12 months (m), 20 m, 41 m, and their OS was 21 m, 26 m, 51 m, respectively. Of the *MYCL1* non-amplification patients, their median DFS were 42 ± 5.82 m, and their median OS were 48 ± 5.22 m, respectively. The survival curves of DFS and OS were shown in Figures 2A,B.

**TABLE 2:** Clinicopathological data of MYCL1 and c-Myc status.

	MYCL1				c-Myc	
	FISH		IHC		IHC	
Factors	Amplified	Non-amplified	Positive	Negative	Positive	Negative
Gender						
Male	2	34	3	33	4	32
Female	1	9	0	10	0	10
Age						
<60	0	24	2	22	2	22
≥60	3	19	1	21	2	20
Smoking						
Non- and light smoker	1	15	0	16	1	15
Moderate and heavy smoker	2	28	3	27	3	27
Stage						
I	1	21	3	19	1	21
II-III	2	22	0	24	3	21
Lymph node metastasis						
N0	1	21	3	19	1	21
N+	2	22	0	24	3	21
Brain metastasis						
No	3	31	3	31	4	30
Yes	0	12	0	12	0	12

### Prevalence of L-Myc Protein Expression by IHC

The L-Myc protein expression was evaluated in 46 SCLC cases**.** The L-Myc protein was located in the nucleus. As shown in Figure 1, case 2 represented positive patient of L-Myc protein expression. Of the evaluated specimens, 6.5% (3 of 46) of specimens were positive for L-Myc protein expression. Of the three positive patients, two patients were less than 60 years old and the other one more than 60 years old. They were all male and moderate and heavy smokers with stage I. No brain metastasis was observed in the above three positive cases during the follow-up (Table 2). Of the three positive patients, two patients were alive, and one patient had died. DFS and OS of the deceased patient were 55 m and 61 m, respectively. Of the L-Myc protein expression negative patients, their median DFS and OS were 39 ± 5.79 m, 46 ± 5.22 m, respectively. The survival curves of DFS and OS were shown in Figures 2C,D.

### Prevalence of c-Myc Protein Expression by IHC

**FIGURE 3:**
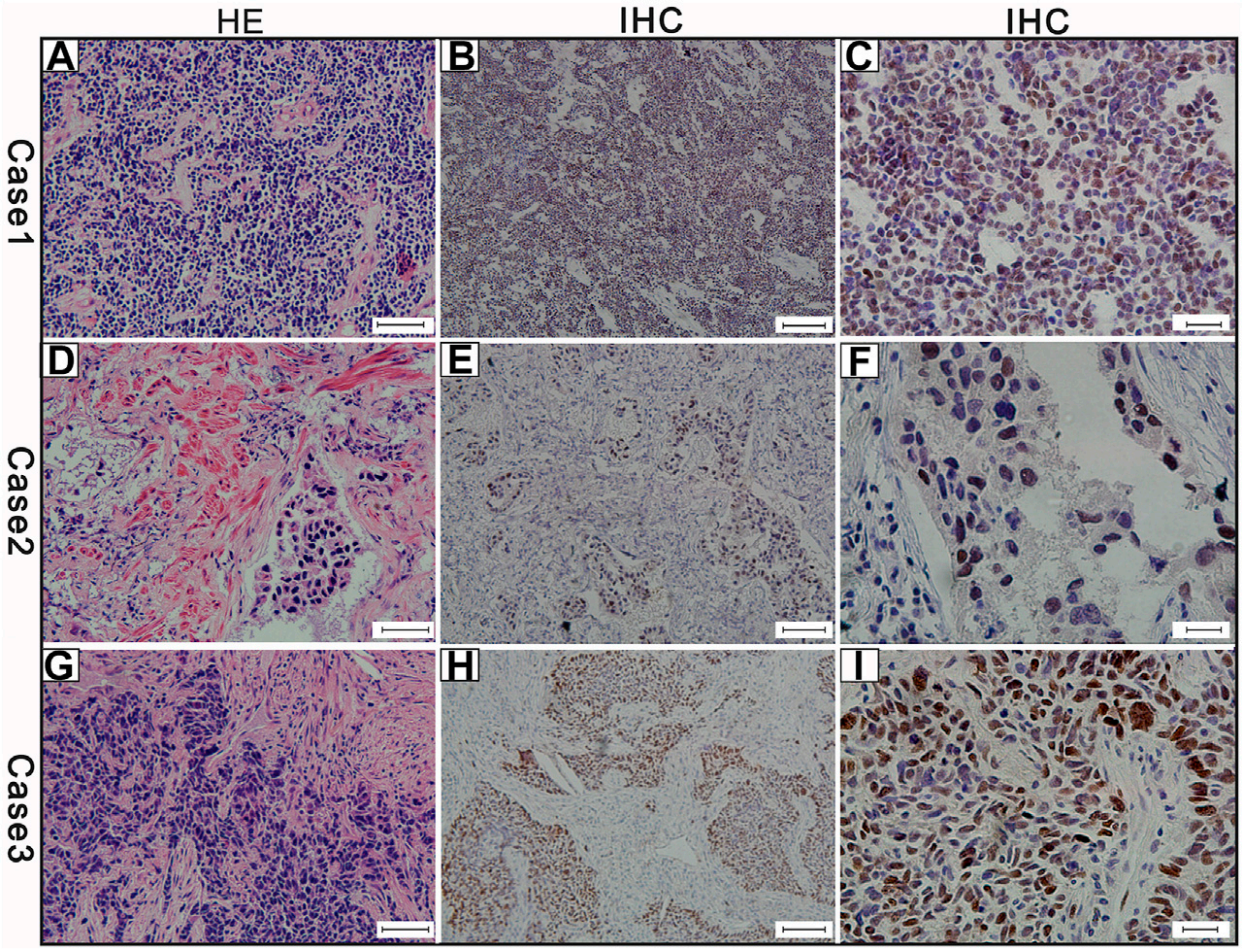
The results of the three cases of c-Myc protein expression **(A)** Hematoxylin and eosin **(H,E)**-staining of case-1 (scale bar 50 μm) **(B)** c-Myc protein positive expression of case-1 (scale bar 100 μm) **(C)** c-Myc protein expression positive of case-1 (scale bar 20 μm) **(D)** Hematoxylin and eosin **(H,E)**-staining of case-2 (scale bar 50 μm) **(E)** c-Myc protein positive expression of case-2 (scale bar 100 μm) **(F)** c-Myc protein expression positive of case-2 (scale bar 20 μm) **(G)** Hematoxylin and eosin **(H**,**E)**-staining of case-3 (scale bar 50 μm) **(H)** MYC protein positive expression of case-3 (scale bar 100 μm) **(I)** MYC protein expression positive of case-3 (scale bar 20 μm). Brown color of the cytoplasm and nucleuses were defined as positive staining.

The c-Myc protein expression was evaluated in 46 SCLC cases**.** The c-Myc protein was located either in the nucleus or both in the cytoplasm as well as the nucleus (Figure 3). Among the evaluated specimens, 8.7% (4 of 46) were positive for c-Myc protein expression. Of the four positive patients, two patients were less than 60 years old and the other two more than 60 years old. They were all male and three of them moderate and heavy smokers and the other one was a light smoker. Three cases were stage at IIIA and one was stage at IA. No brain metastasis was observed in the above three positive cases during the follow-up (Table 2). Of the four positive patients, all patients were alive. Of the c-Myc protein expression negative patients, their median DFS and OS were 35 ± 4.90 m, 42 ± 4.35 m, respectively. The survival curves of DFS and OS were shown in Figures 2E,F.

### Correlation of L-Myc Protein Expression with *MYCL1* amplification and Fusion

In all 46 SCLC cases, IHC and FISH were performed successfully, the *MYCL1* FISH-positive in 3 cases, and L-Myc IHC-positive in 3 cases. However, there was no overlap between L-Myc protein expression and *MYCL1* amplification and fusion.

### Correlation of *MYCL1* amplification, L-Myc Protein and c-Myc Protein Expression

In all 46 SCLC cases, 4 c-Myc-positive cases had no overlap with 3 *MYCL1* FISH and 3 L-Myc protein positive cases. No correlations between *MYCL1* amplification, L-Myc protein expression and c-Myc protein expression were found.

## Discussion

Little is known about C-MYC and MYCL1 in resected SCLC samples due to rare surgical resection in SCLC. For the first time, postoperative specimens were used in this study to comprehensively analyze *MYCL1* amplification, L-Myc and c-Myc protein expressions. Among the 46 evaluated specimens, *MYCL1* amplification was identified in 3 of 46 cases (6.5%). Interestingly, one of the three positive cases simultaneously had *MYCL1* amplification and fusion, which need further explored. 3/46 (6.5%) specimens were positive for L-Myc protein expression and 4/46 (8.7%) of the specimens were positive for c-Myc protein expression.

The MYC family has been reported to be amplified in a subset of SCLCs. Recent studies have indicated that C-MYC and MYCL1 may be the potential targets for SCLC, which could modulate the tumor microenvironment and intratumoral heterogeneity in SCLC *in vivo* and *vitro* [18, 19]. As had been proved, MYCL1 was tightly related to neuroendocrine cancers, such as SCLC and Merkel cell carcinoma [12, 20]. From all above, C-MYC and MYCL1 play crucial roles in the malignant behaviors of SCLC.

In our cohort, *MYCL1* amplification was observed in 6.5% SCLC cases using FISH, and L-Myc protein expression was observed in 6.5% of SCLC cases. So far, our study is the first research focus on *MYCL1* amplification in SCLC. The prevalence of *MYCL1* amplification was in accordance with the data from the genomic profiles of SCLC [5]. However, there was no relation between L-Myc protein expression and high *MYCL1* gene copy number in our study although the underlying mechanism was undetermined.

According to IHC results, c-Myc protein expression was identified in 8.7% (4/46) of SCLC patients in our study, which was lower than the results of the following study. It has been reported that 38% (39/103) of SCLC patients showed some degree of c-Myc protein expression. *MYC* amplification by CISH was observed in 9.3% (9/97) of SCLC patients, and was correlated with protein expression [7]. Another study suggested that *MYC* amplification by CISH was detected in 20% (11/55) of SCLCs [21]. Our study only detected c-Myc protein expression, not *MYC* amplification, so we were unable to confirm the correlation between the c-Myc protein expression and *MYC* amplification. In addition, in terms of differences of c-Myc protein expression, a possible explanation to the discordance might be related to the different disease stage and specimens used. Of 46 SCLC cases in our study, all were larger specimens and all cases were resected with stage I–III, not advanced metastatic SCLC. However, in the other study, 103 SCLC cases with stage I–IV (50% cases were extensive-stage) were included [7]. The above results suggested that c-Myc protein expression may be affected by stage.

However, whether C-MYC and MYCL1 affect the survival remains unascertained in SCLC. Hwang et al*.* found no statistical relationship between *MYC* amplification and OS in the untreated SCLC patients (50% extensive-stage) [7]. Alves RC et al. reported *MYC* amplification was associated with poor survival (4.67 weeks of patients with *MYC* amplification vs. 26.15 weeks of patients without *MYC* amplification, *p* = 0.02) in the untreated SCLC patients [22]. It is worth noting that about 85% of the above SCLC patients were in an advanced stage with a median OS of 22.91 weeks (5.7 months), that was shorter than the usual median OS (about 10 months). In addition, their 20% *MYC* amplification prevalence was higher than the usual rate (about 7%). A possible explanation to this discordance might be that a mean of 60 cells were evaluated, which influenced the statistical results. In a study conducted by Chen et al, multivariate analysis results showed that L-Myc expression was an independent prognostic factor of gastric cancer, which suggested that L-Myc expression is a useful biomarker for gastric cancer prediction and a promising therapeutic target for gastric cancer treatment [11]. Additionally, colorectal cancer patients whose tumors exhibited LOH at *MYCL1* on chromosome 1p34 show poor prognosis, which indicated that *MYCL1* may be used as a biomarker with clinical relevance [23]. *MYCL1* activation might be associated with efficacy and disease relapse. Recent work has shown that longitudinal cell-free DNA (cfDNA) analysis in SCLCs played important roles in revealing dynamic insights into the treatment efficacy and disease relapse. Profiling of patient’s cfDNA prior to first-line chemoradiotherapy revealed *MYCL1* amplification (15.5 copies). Following treatment with cisplatin and etoposide, no decrease of amplification level was detected in all serial samples collected up to 413 days after diagnosis, which suggested that the patient had platinum-sensitive disease [22]. The impact of *MYCL1* and C-*MYC* on survival may be too small to be detected. Alternatively, the sample size and baseline differences may also have a bias on outcomes. Therefore, the results are not necessarily objective and reliable. There is another possibility that these biomarkers ultimately maybe contribute to predicting the treatment response in SCLC, rather than to predicting prognosis.

Methods have been found to modulate Myc family pathways through targeted therapy. However, directly targeting Myc family for therapy is still elusive due to lack of a well-established ligand-binding domain. Therefore, much attention has been paid on the targeting downstream regulators. Aurora kinase inhibitor alisertib is hypothesized to be a target of *MYCL1* downstream pathway, further focusing on the investigation of *MYCL1* fusions in SCLC. Silencing *MYCL1* in SCLC cell lines with *RLF-MYCL1* fusion results in the decreased cell proliferation [24]. A few SCLC patients may benefit from alisertib, showing promising results in clinic trials [25, 26]. A total of 689 SCLC patients were assayed with hybrid-capture based comprehensive genomic profiling (CGP). *MYCL1* amplification was identified in 53 cases, and six were *MYCL1* fusions (*MYCL1-COL9A2*, *MYCL1-MSRB2*, *MYCL1-PABPC4*, *MYCL1-MACF1*, *MYCL1-JAZF1*, and one with indeterminate partner). All arose from inter-chromosomal rearrangements. A non-smoker SCLC patient (aged 46 years, male) was identified to harbor *MYCL1-JAZF1*, and had near complete response to alisertib for 18 months following the failure of three previous lines of chemotherapy [25]. Further investigation regarding *MYCL1* fusions in SCLC patients is warranted to assess possible functions as oncogenic drivers. Considering the positive effect of alisertib or other therapies targeting MYCL1 pathway, the ability to identify MYCL1 protein expression and amplification or/and fusion might be an essential component of pathological workup. *MYCL1* fusion was detected using FISH, which was present in 6.5% of SCLC patients in this study. However, the IHC results were negative. The status and correlation of MYCL1 fusion and expression in SCLC patients in China are still unknown. Hence, in this paper, *MYCL1* fusion and expression were detected in SCLC patients to serve as the basis for precision treatment. Besides, it is noticed that there are abundant lymphocytes infiltrating in the mesenchyme in six cases (L-Myc positive and *MYCL1* amplification). Lymphocyte infiltration was closely related to local immune response, which can be used as a biomarker of immunotherapy. It has been reported that MYCL1 selectively expressed in the dendritic cells play an important role in infection immunity and is required for optimal T-cells priming following bacterial and viral infection. Does MYCL1 expressed by cancer cells also activate T-cells to mediate tumor immunity? How does the interaction between MYCL1 and T-cell interference influence on SCLC patient outcomes? These questions are interesting and considerable.

However, our study has some limitations. Although the sample size of our study on postoperative specimens for SCLC is the relatively large, it is still insufficient and the MYCL1-and MYC-positive samples remain few. The significant differences between the clinical characteristics and outcomes of patients with positive and negative groups were not enough to statistical identified. Therefore, we yielded to evaluate clinical characteristics and survival analysis, because the low number of positive cases does not permit reliable evaluation. To identify such factors, a larger sample size of SCLC patients is needed.

In conclusion, our findings from the present study showed that *MYCL1* and MYC were infrequently detected in the resected SCLCs. Our study provide information for detection, clinicopathological characteristics, and survival of MYCL1 and c-Myc positive patients with surgically resected SCLC, which makes a contribution to subsequent research and therapeutic strategies. Additional correlative studies with larger cohort are needed to determine whether the protein expression or gene amplification is more predictive and/or has prognostic impact on Myc pathway dependency.

## Data Availability

The original contributions presented in the study are included in the article, further inquiries can be directed to the corresponding author.
